# Editorial: Biomarkers and precision medicine in myeloid neoplasms

**DOI:** 10.3389/fcell.2026.1947937

**Published:** 2026-08-26

**Authors:** Ananya Datta Mitra

**Affiliations:** Department of Pathology and Laboratory Medicine, University of California Davis, Sacramento, CA, United States

**Keywords:** biomarkers, mastocytosis, myeloid neoplasm, precision medicine, therapy related myeloid neoplasm

## Introduction

Cancer evolution is commonly believed to occur through a gradual, stepwise process driven by molecular diversification and the clonal expansion of neoplastic stem cells (Nowell, 1976; Vogelstein and Kinzler, 1993). Myeloid neoplasms including acute myeloid leukemia (AML), myelodysplastic neoplasms (MDS), myeloproliferative neoplasms (MPNs), and myelodysplastic/myeloproliferative neoplasms (MDS/MPN) and mastocytosis are heterogeneous group of neoplasms affecting the pluripotent hematopoietic precursor cells residing in the myeloid compartment of the bone marrow.

Over the past decade, both predictive and prognostic biomarkers have become central to diagnostics, classification, prognosis, and targeted treatment for this diverse group of neoplasms, improving efficacy and reducing side effects in these patients. Recent advances in molecular techniques like next-generation sequencing (NGS), transcriptomics, proteomics, and single-cell technologies have modified our interpretation of the molecular background of myeloid neoplasms. These revolutions have led to the identification of unique biomarkers, including recurrent gene mutations (e.g., FLT3, IDH1/2, and TP53), epigenetic alterations, and immune-related signatures that deliver valuable insights into disease biology. Such discoveries help to improve the risk stratification, advance diagnostic and prognostic precision, and facilitate the development of tailored therapeutic strategies, including FLT3 inhibitors, IDH inhibitors, and BCL-2 antagonists (Döhner et al., 2022; Aziz et al., 2017). Our research topic, “Biomarkers and Precision Medicine in Myeloid Neoplasms,” was designed to address this rapidly advancing landscape and, essentially, to examine how molecular discoveries can be translated into clinical practice, ultimately improving diagnosis, risk assessment, treatment selection and outcomes for patients with myeloid neoplasms. Seven articles were eventually accepted for publication, covering translational investigations, molecular studies, retrospective clinical analyses, and a diagnostically challenging case. While these studies address discrete myeloid neoplasms and biological questions, collectively they contribute to several consistent themes like the expansion of the biomarker repertoire beyond recurrent mutations, refinement of diagnostic and prognostic assessment, better understanding of disease progression and therapeutic resistance, and the importance of integrating molecular findings with clinical, morphologic, and cytogenetic information.

## Expanding the biomarker landscape beyond recurrent genetic mutations

One crucial contribution of this Research Topic is the demonstration that clinically significant biomarkers extend beyond standard recurrent driver mutations. Increasingly, the field is advancing toward a more comprehensive view of disease biology in which protein expression, epigenetic alterations, and molecular signatures balance genomic findings.

The original article by Silva B et al. “*Reduced STAG2 expression in myelodysplastic neoplasms and acute myeloid leukemia myelodysplasia-related: a potential biomarker associated with aneuploidy and disease progression*”, explores the clinical and biological implication of STAG2 protein expression in myelodysplastic neoplasms (MDS). While mutations in *STAG2*, a core component of the cohesin complex, have been well recognized as recurrent genetic events in MDS, the clinical implications of STAG2 protein expression have remained largely unelucidated. By assessing STAG2 expression in bone marrow biopsies from pediatric and adult patients with MDS, the authors demonstrate that reduced STAG2 protein expression is strongly associated with cytogenetic abnormalities, particularly complex karyotypes and trisomy 8, both of which are linked to adverse clinical outcomes. These findings demonstrate the relevance of *STAG2* beyond mutation status and imply that assessment of protein expression may provide supplementary information regarding genomic instability and disease progression.

A similar expansion of the molecular landscape is demonstrated by Kasmi et al. “*Molecular characterization of pediatric mastocytosis revealed different somatic mutations with uncertain prognostic value*”, explores the molecular landscape of pediatric mastocytosis, a rare clonal mast cell disorder for which the genetic basis remains considerably less understood than in adult disease. While activating KIT mutations, particularly KIT D816V, are well-established drivers of systemic mastocytosis in adults, the molecular heterogeneity and clinical relevance of additional genetic alterations in pediatric mastocytosis have yet to be fully explored. By combining highly sensitive molecular techniques, including reverse transcription PCR (RT-PCR), droplet digital PCR (ddPCR), and targeted next-generation sequencing (NGS), the authors provide one of the most comprehensive molecular characterizations of pediatric mastocytosis reported to date.

The study establishes that KIT mutations are present in approximately one-third of pediatric patients, while also identifying several additional mutations involving genes implicated in myeloid neoplasia, including ASXL1, TET2, SH2B3, JAK2, ETV6, and NFE2. Notably, several of these variants have not been described in pediatric mastocytosis in the past, expanding the current interpretation of the disease’s genetic landscape.

Collectively, these studies demonstrate an expanding shift in biomarker research from discovering individual driver mutations toward understanding multifaceted molecular phenotypes that incorporate genomic, protein, and other biological alterations.

## Identifying disease evolution and mechanisms of therapeutic resistance

Another notable knowledge gap addressed by this Research Topic encompasses the evolving nature of myeloid neoplasms. Molecular biomarkers may not only identify disease at diagnosis but may also provide insight into how disease develops following exposure to cytotoxic therapy and how malignant cells adapt and modify to treatment.

The third original resarch article by Uekusa et al. “*Real-world data-based assessment of therapy-related myeloid neoplasms after poly(ADP-ribose) polymerase inhibitor treatment in ovarian cancer*”, showcased a real-world analysis examining the incidence and potential predictors of therapy-related myeloid neoplasms (t-MNs) among patients with ovarian cancer treated with poly(ADP-ribose) polymerase inhibitors (PARP inhibitors). Although these agents have transformed the management of BRCA-mutated and homologous recombination-deficient ovarian cancers, the emergence of t-MNs represents a rare but potentially life-threatening adverse event that warrants careful surveillance. In a retrospective cohort of patients treated across two institutions, the authors identified t-MNs in a small proportion of patients, all of whom had received various platinum-based chemotherapy regimens and subsequently developed myeloid neoplasms 5 years after their initial cancer diagnosis. While no statistically significant clinical or hematologic predictors of t-MN development were recognized, the study observed a trend toward patients with higher body mass indices and with greater declines in white blood cell counts following PARP inhibitor initiation who later developed t-MNs. Even though these findings require confirmation in larger cohorts, they emphasize the challenges of identifying patients at risk using routine clinical and laboratory parameters.

These findings emphasize an important unsolved question in therapy related myeloid neoplasms: which patients harbor susceptibility to clonal evolution, and how can this risk be diagnosed before overt disease develops? The absence of consistent clinical predictors highlights the need for future studies incorporating clonal hematopoiesis, germline susceptibility, serial molecular profiling, and longitudinal blood count changes.

## Modifying prognostic assessment through genomic and epigenetic information

Another major theme emerging from the Research Topic is the potential for molecular biomarkers to improve prognostic assessment beyond established clinical and cytogenetic parameters. This is particularly important in myeloid neoplasms such as AML and MDS, in which patients with evidently similar clinical or cytogenetic characteristics can experience markedly distinct outcomes.

The original research published by Liu et al. “*Clinical perception and novel insights of chronic myelomonocytic leukemia: a 10-year multi-center retrospective study*”,addresses the knowledge gaps in clinical heterogeneity and prognostic complexity of chronic myelomonocytic leukemia (CMML), an overlap neoplasm with myelodysplastic and myeloproliferative features. Despite advances in the molecular characterization of CMML, its relatively low incidence and biological diversity continue to present significant challenges in diagnosis, risk stratification, and therapeutic management. Through a large multicenter cohort, the authors provide effective and practical insights into the clinical characteristics, treatment patterns, and outcomes of patients with CMML, contributing important evidence to supplement modern clinical practice.

Furthermore, Zhang et al. in their original Reaserch article, “*Identification and validation of HOXB3 hypomethylation as a novel prognostically epigenetic biomarker in acute myeloid leukemia*”, the investigators explore the prognostic significance of DNA methylation within the HOXB gene family, extending previous work on HOXA methylation to further explain the epigenetic landscape of AML. Their study identify HOXB3 hypomethylation as a recurrent epigenetic alteration linked with increased gene expression and adverse clinical outcomes in biologically distinct subsets of AML, including cases with normal karyotype, intermediate-risk disease, and recurrent mutations in FLT3-ITD, NPM1, and DNMT3A. This study highlights the growing potential of DNA methylation profiling as a complementary approach to genomic testing for modifying prognostic assessment and advancing precision medicine in AML.

Altogether, these studies emphasize that precision medicine requires more than identifying molecular abnormalities; it requires determining how those abnormalities improve risk assessment in clinically diverse patient populations. They also highlight the need to validate emerging biomarkers across independent cohorts before they can be integrated into routine clinical algorithms.

Tiedemann et al. in their original reaserch, “*Association of fibronectin 1 deregulation with tyrosine kinase inhibitor resistance in chronic myeloid leukemia*”, the authors examine the role of fibronectin 1 (*FN1*), an extracellular matrix protein traditionally associated with tumor invasion and metastasis in solid malignancies, as a novel regulator of tyrosine kinase inhibitors (TKIs) response in CML. The research establishes that *FN1* expression is down-reguated in TKI-resistant CML cell models *in vitro*. Furthermore, the loss of *FN1* reduces sensitivity to multiple generations of BCR::ABL1 inhibitors, including asciminib and that restoration of *FN1* expression re-sensitized resistant cells to TKI therapy, including models harboring clinically relevant BCR::ABL1 kinase domain mutations such as *E255K* and the gatekeeper mutation *T315I*. These findings broaden our knowledge of the complex mechanisms underlying TKI resistance and that FN1 can serve as an alternate target in patients with TKI resistance.

## Molecular testing as an essential component of diagnostic integration

The ultimate major theme emanating from the Research Topic is the perpetual importance of integrating molecular biomarkers with morphology and conventional pathology rather than viewing molecular testing as a proxy for established diagnostic approaches.

The final published article, from Zhang et al. “*The appearance of “faggot Auer rods” in blasts of non-M3 acute myeloid leukemia*” describe a rare case of acute myeloid leukemia (AML) showing striking “faggot-like” Auer rod aggregates, a distinct cytomorphologic feature characteristically associated with acute promyelocytic leukemia (APL). Despite the typical morphology and strong myeloperoxidase staining, comprehensive cytogenetic, and molecular investigations excluded the presence of *PML::RARA* and other APL-defining fusion genes, rendering the diagnosis of a non-APL subtype of AML. This case strongly emphasizes that even characteristic morphologic findings may not be disease specific and that molecular confirmation remains essential for an accurate diagnosis. Collectively, these contributions reflect the multidisciplinary and rapidly evolving environment of biomarker research in myeloid neoplasms. They focus the need for continued association amongst clinicians and pathologists with advancing molecular technologies to validate novel biomarkers, standardize their implementation, and integrate multiomics technologies into routine clinical care.

Taken together, the seven contributions help elaborate several unmet needs in the advancing field of myeloid neoplasms. First, they establish that the biomarker landscape extends beyond recurrent mutations to include protein expression, epigenetic alterations, gene-expression changes, and extracellular or cellular related factors. Second, they highlight that discovery alone is inadequate; biomarkers must be critically validated and clinically correlated with outcomes before they can be meaningfully implemented in patient care. Third, they emphasize that biomarkers must be interpreted within the dynamic context of clonal evolution and treatment exposure. Finally, they strengthen the pivotal role of multidisciplinary integration, in which molecular findings are interpreted together with morphology, cytogenetics, immunophenotype, and clinical information.
